# Evaluation of Matrix-Assisted Laser Desorption/Ionization Time-of-Flight (MALDI-TOF) Mass Spectrometry for Identification of Adult *Schistosoma mansoni* Worms and Eggs

**DOI:** 10.3390/pathogens15050534

**Published:** 2026-05-15

**Authors:** Lucie Conrad, Franco H. Falcone, Sören L. Becker, Issa Sy

**Affiliations:** 1 Institute of Medical Microbiology and Hygiene, Saarland University, 66421 Homburg, Germany; lucie.conrad@gmx.de (L.C.); soeren.becker@uks.eu (S.L.B.); 2 Institute of Parasitology, BFS, Justus Liebig University Giessen, 35392 Giessen, Germany; franco.falcone@vetmed.uni-giessen.de; 3 Helmholtz Institute for Pharmaceutical Research Saarland, 66123 Saarbrücken, Germany; 4 PharmaScienceHub, 66123 Saarbrücken, Germany

**Keywords:** *Schistosoma mansoni*, trematodes, helminths, matrix-assisted laser desorption/ionization time-of-flight (MALDI-TOF) mass spectrometry, identification

## Abstract

Schistosomiasis, a neglected tropical disease (NTD), affects humans and leads to considerable clinical morbidity and severe long-term sequelae. Laboratory diagnostics for *Schistosoma mansoni* are mainly based on microscopic identification of eggs in stool, but sensitivity varies with infection intensity. Matrix-assisted laser desorption/ionization time-of-flight (MALDI-TOF) mass spectrometry (MS) is the gold standard for bacterial identification in high-income countries. Here, we first evaluate the capacity of MALDI-TOF MS and our existing ‘in-house helminths’ database for the identification of *S. mansoni* worms and eggs. A subset of adult worms and egg samples was used to generate MALDI reference spectra, which were added to the database and evaluated by blind-test identification. Subsequently, egg-free human stool was spiked with purified *S. mansoni* eggs and analyzed by MALDI-TOF MS. Log score values (LSVs) were employed to assess the reliability of identification. A total of 62/90 (68.9%, 95% confidence interval (CI): 58.3–78.2%) adult samples were correctly identified. After database expansion, 90/90 (100%, 95% CI: 96.0–100%) and 59/60 (98.3%, 95% CI: 91.1–100%) were correctly identified for adult worms and purified eggs, respectively. In contrast, the analysis of 35 human stool samples spiked with *S. mansoni* as eggs did not yield identifiable spectra. MALDI-TOF MS can be applied for the identification of isolated adult *S. mansoni* worms and eggs. Further investigations and optimization are necessary before potential application to clinical samples (e.g., for egg detection in stool).

## 1. Introduction

Schistosomiasis (bilharzia) is a neglected tropical disease (NTD) [1], that infects over 250 million people worldwide but is only endemic in specific regions [2,3]. Several species and hybrid species affecting humans have been described, the most important of which are *Schistosoma mansoni*, *Schistosoma japonicum* and *Schistosoma haematobium* [3,4,5]. Animals can also develop schistosomiasis (e.g., *Schistosoma bovis* can cause intestinal lesions and granuloma in different organs leading to appetite loss, enteritis, anemia and anorexia in ruminants [4]). These parasites are also of zoonotic significance as hybrid species like *S. haematobium* x *S. bovis* exist and highly pathogenic species (e.g., *S. japonicum*) can be transmitted from animal hosts to humans. Domestic ruminants or pigs can serve as pathogen reservoirs in endemic areas [4,5]. Contact with cercariae-contaminated freshwater is required to complete the life cycle. After penetration of the hosts’ skin, schistosomula migrate via the lungs to the portal vein and become mature. Adult worms can then be found in the host veins, where the female worm produces eggs, that are then excreted either in the urine (*S. haematobium*) or with the feces (e.g., *S. mansoni*, *S. japonicum*). While *S. haematobium* is found in veins around genital organs, *S. mansoni* and *S. japonicum* are found in mesenteric veins [3]. Clinical symptoms of schistosomiasis are manifold. The penetration of cercariae (mostly *S. japonicum*) into the human skin can trigger cercarial dermatitis. Furthermore, *Schistosoma* eggs can initiate development of granulomas in multiple organs. *S. haematobium* can cause inflammation and obstructive lesions as well as ulcers in the urinary tract. *S. haematobium* eggs cause grainy as well as homogenous sandy patches in female genital tissue. Symptoms can include bladder dysfunction hematuria and mucosal bleeding. Also, severe long-term sequelae such as bladder cancer may arise [3,4,5,6,7,8]. In the case of *S. mansoni* and *S. japonicum* intestinal symptoms like diarrhea, appetite loss, inflammation of spleen, gall bladder and liver, and even liver fibrosis may arise [7,9].

Diagnostic methods are based on microscopic detection of eggs in urine or feces, but sensitivity is limited, as it has been reported as 4% for the detection of *S. haematobium* in urine and 33.7% for the detection of *S. mansoni* in stool [10]. The Kato–Katz technique (thick smear microscopy) is widely used for diagnosis of intestinal schistosomiasis but lacks sensitivity in low-infection intensity cases. PCR techniques with higher sensitivity have also been developed [10,11], but are still time-consuming, and expensive for routine applications. PCR methods have a sensitivity of 10.5% for detection of *S. haematobium* in urine and 48.8% for detection of *S. mansoni* in stool. Furthermore, a sensitivity of 72.7% and specificity of 98.9% was reached for PCR in serum samples [10]. Sensitivities of 88% and 95% for *S. mansoni* detection using circulating cathodic antigens (CCAs) and ELISA tests, respectively, have been reported in comparison with parasitological stool examinations used as reference, while specificities were 72% and 35%, respectively [12].

Matrix-assisted laser desorption/ionization time-of-flight (MALDI-TOF) mass spectrometry (MS) is nowadays the gold standard diagnostic tool for species identification of culture-grown bacteria in clinical microbiology laboratories in high-income countries. It is a rapid, reliable and cost-effective technique allowing us to generate species-specific protein mass spectra profiles, which can rapidly be compared to species-specific reference spectra in a database. The commercially available species identification database includes spectra of bacteria, fungi and mycobacteria. For sample analysis, a small portion of the specimen is mixed with a matrix before being deposited on a target plate and placed in the mass spectrometer [13,14,15,16,17]. More recently, some studies have evaluated MALDI-TOF MS for arthropods, e.g., for ticks [18] and mosquitoes [19,20]. Further studies have analyzed the capacity of MALDI-TOF MS to diagnose parasitic helminths [21] such as cestodes [22], trematodes [23] and nematodes [24,25,26,27]. We recently reported the application of MALDI-TOF MS for identification of adult *Schistosoma* spp. worms [28]. Here, the primary objective was to evaluate whether MALDI-TOF MS can serve as a reliable complementary diagnostic tool for the identification of adult *S. mansoni* worms and eggs and assess its potential applicability in parasitological diagnostics. Specifically, we hypothesized that adult *S. mansoni* worms and eggs (isolated eggs and spiked egg-positive stools) generate reproducible, species-specific protein spectra that can be accurately identified using a MALDI-TOF MS reference database.

## 2. Materials and Methods

### 2.1. Sample Collection

Adult *S. mansoni* worms and eggs (Naval Medical Research Institute (NMRI) strain) were provided by the ‘Schistosomiasis Resource Center of the BRI (Rockville, MD, USA)’, where they were collected from livers and intestinal tissues of experimentally infected mice in November 2023 [29]. All samples were frozen and sent to the Institute for Medical Microbiology and Hygiene in Homburg, Germany. Upon arrival in April 2024, samples were stored at −20 °C until analysis. As a reference laboratory, the BRI ensured the bona fide identity of the material as well as the determination of the sex, based on controlled life-cycle maintenance and expert identification. Given this controlled origin, additional genetic characterization was not performed in the present study. Human stool samples used for spiking originated from our local biobank and previously collected from a Schistosoma-endemic region (Cote d’Ivoire), were classified as egg-negative based on the duplicate Kato–Katz thick smear method.

### 2.2. MALDI-TOF MS Analysis

#### 2.2.1. Protein Extraction of Adult Schistosoma Mansoni Worms

For each sample, 2 adult worms were placed into a 1.5 µL Eppendorf tube. The sex composition of the samples was defined as follows: male-only samples (two male worms), female-only samples (two female worms), mixed samples (one male and one female worm). Protein extraction was conducted according to a previously developed protocol [23]. In detail, 300 µL water (HPLC grade) and 900 µL absolute ethanol were added to the sample (containing 2 adult worms) and mixed by vortex for 1 min. After centrifugation at 18,312× *g* for 2 min, the supernatant was discarded, and the sample was dried under the biosafety cabinet. Twenty µL of 70% formic acid (*v*/*v*) and 20 µL of acetonitrile were added and vortexed, followed by a final centrifugation step at 18,312× *g* for 2 min.

#### 2.2.2. Protein Extraction of Schistosoma Mansoni Eggs

For protein extraction of the isolated *S. mansoni* eggs, three different protocols were applied to the samples (each tube containing approximately 1000 eggs, as provided by the Schistosomiasis Resource Center of the BRI). Protocol 1 consisted of dilution of the eggs in 1.2% cold NaCl solution to obtain three different concentrations. Concentration 1 (C1) contains 20 eggs/µL, C2 corresponds to 10 eggs/µL, and C3: 5 eggs/µL. Protocol 2 involves C1 (50 eggs/µL), C2 (25 eggs/µL) and C3 (12.5 eggs/µL). Each tube was centrifuged (18,312× *g*, for 2 min). After discarding the supernatant, a pinch of glass beads (BioSpec Products, Carl Roth GmbH+Co. KG, Karlsruhe, Germany) was added, as well as 20 µL of 70% formic Acid (*v*/*v*), and 20 µL acetonitrile. Samples were then homogenized using the FastPrep™-24 5G machine (MP Biomedicals™, Irvine, CA, USA), followed by a final centrifugation at 18,312× *g* for 2 min. In Protocol 3, eggs were directly added in 100 µL formic acid 70% (*v*/*v*) and 100 µL acetonitrile. After adding glass beads to the sample, the same steps as for protocol 1 and protocol 2 were followed.

#### 2.2.3. Target Plate Preparation and Measurements

From each sample, 1 µL of the clear supernatant was placed on eight different spots of the MALDI-TOF target plate. After the air-drying of the spots, 1 µL of a-Cyano-4-hydroxycinnamic acid matrix solution (HCCA) (Bruker Daltonics, Bremen, Germany) was added to each spot, and each spot was measured four times. All samples were measured using the Microflex LT Mass Spectrometer (Bruker Daltonics, Germany). In total, 32 raw spectra were generated for each sample. For calibration of the machine, a bacterial test standard (BTS) (Bruker Daltonics, Germany) was used.

#### 2.2.4. MALDI-TOF MS Parameters

Spectra were acquired with the FlexControl^®^ software version 3.4 (Bruker Daltonics, Germany) using the AutoXecute algorithm. For each spot, 240 laser shots were recorded in six random positions in a linear positive ion mode with a laser frequency of 60 Hz, a high voltage of 20 kV, and a pulsed ion extraction of 180 ns. Measured mass-to-charge ratios (*m*/*z*) comprised between 2 k and 20 kDa.

#### 2.2.5. Spectral Analysis and Database Creation

The FlexAnalysis^®^ software version 3.4 (Bruker Daltonics, Germany) was used for editing the raw spectra. Aforementioned spectra were subjected to preprocessing steps such as baseline subtraction, smoothening of the intensities, removal of flatlines and outlier peaks. In accordance with previous studies suggesting a need for ≥10 reference spectra to obtain accurate MALDI-TOF identification [30], a total of 12 adult and 11 egg samples with at least 20 replicated spectra each were selected for creating average species-specific main spectra profiles (MSPs) to be used as reference spectra for database expansion. Newly created MSPs were then added to the existing in-house database (containing *Schistosoma mansoni* spectra from adult male and female worms (Puerto Rico strain, obtained from experimentally infected mice in the Laboratory of Tropical Medicine and Parasitology of Dokkyo Medical University, Tokyo, Japan [28])) by using the automatic function of the MALDI Biotyper Compass Explorer^®^ software version 4.1.9 (Bruker Daltonics, Germany). A dendrogram analysis (hierarchical clustering) of adult MSPs was performed using MALDI Biotyper Compass Explorer^®^ with the following parameters: distance correlation, average linkage, and score threshold values of 300 and 0 (arbitrary unit) for a single and related organism.

#### 2.2.6. Validation Tests

Before validation, the spectra were tested for bacterial or fungal contamination by comparing them to the commercially available database by Bruker Daltonics (BDAL, Bruker Taxonomy, MBT Compass Explorer software version 4.1.90). The validation steps were then conducted with the use of the MBT Compass Explorer^®^ software version 4.1 (Bruker Daltonics, Germany). First, raw spectra of the samples that were used for MSPs creation were compared to the previously installed database. Next, a second validation was performed by analyzing spectra from a subset of new, independent samples submitted to the newly extended database for identification. Ninety adult *S. mansoni* (30 females, 30 males, and 30 mixed samples), and 60 *S. mansoni* eggs were analyzed using the protein extraction protocol described above for adults and protocol 2 for eggs, respectively. Per sample, 4 raw spectra were generated and aligned with the newly extended database. A log score value (LSV) of ≥1.7 was considered to indicate a reliable identification of the sample at the genus level, and an LSV of ≥2.0 indicated a reliable identification of the sample at the species level. The total of raw spectra used for the analysis amounted to 355 for adult worms and 240 for eggs.

#### 2.2.7. Analysis of Stool Samples Spiked with Schistosoma Mansoni Eggs

Human stool samples stemming from our local biobank and classified as free of helminthic infection based on duplicate Kato–Katz thick smear were pooled and distributed in different tubes, each containing 0.1 g of stool. Thirty-five purified egg samples were chosen for this analysis. Protocol 2 (see Section 2.2.2) was applied to 30 of them, and the resulting egg suspensions were spiked into the helminth egg-free stool samples. Subsequently, 1.4 mL of 1.2% NaCl solution was added to the egg-stool mixture. Five other purified egg samples were directly spiked into the negative stool without dilution. A total of 40 egg-free stool samples were used as negative controls. A filtration step using a 200 µm filter, followed by centrifugation at 800× *g* for 5 min, was performed. After removing the supernatant, the same procedure was repeated twice. The resulting sediment was subjected to MALDI-TOF analysis. For this purpose, 100 µL of 70% (*v*/*v*) formic acid and 100 µL acetonitrile, as well as glass beads, were added to the samples. After fragmentation using the FastPrep™-24 5G machine (MP Biomedicals™, Irvine, CA, USA), centrifugation at 18,312× *g* for 2 min, was performed. MALDI-target plate preparation and measurements were conducted as described in Section 2.2.3 and Section 2.2.4.

## 3. Results

### 3.1. MALDI-TOF MS Analysis

#### 3.1.1. Spectra Visualization of MSPs and Clustering Analysis

**Figure 1:**
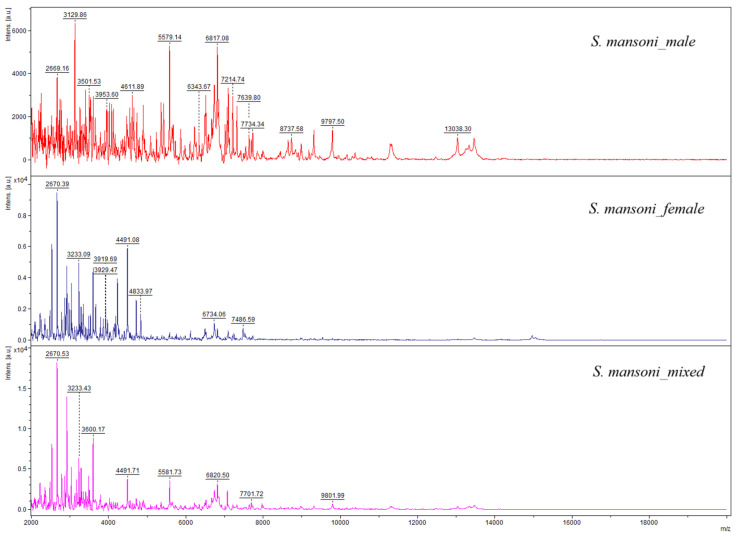
Representative MALDI-TOF spectra of adult *Schistosoma mansoni*.

**Figure 2:**
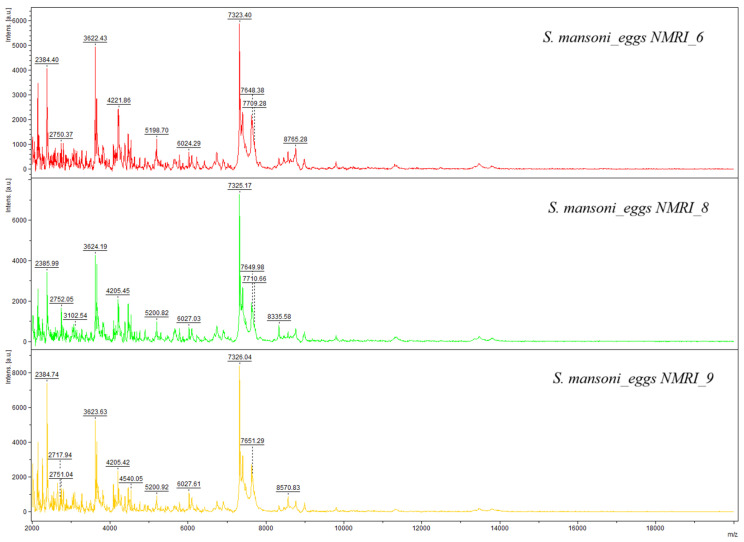
Representative MALDI-TOF spectra of *Schistosoma mansoni* eggs.

**Figure 3:**
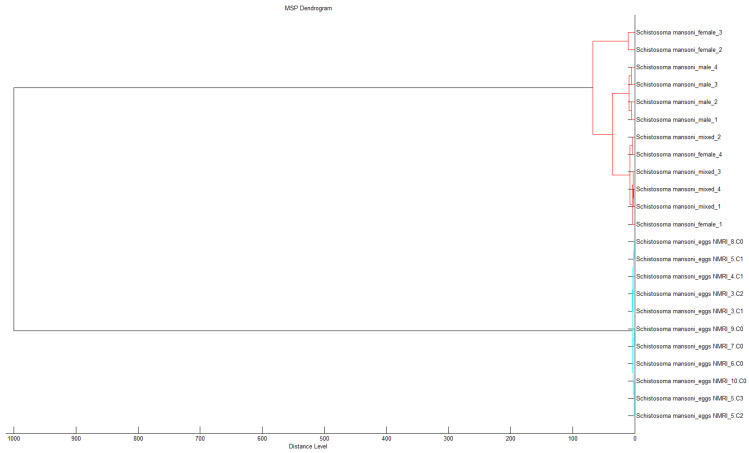
Dendrogram analysis displaying the relatedness of the different *S. mansoni* samples (male adults, female adults, mixed adults, eggs).

Graphical representations of the spectra show in Figure 1, the spectra of adult *S. mansoni*, and in Figure 2, those of *S. mansoni* eggs. Visual differences observed between the spectra profiles of adult worms and eggs were confirmed by a dendrogram analysis, which highlights distinct groups, differentiating between adult worms and eggs. Clustering confirms the specificity of the spectra profiles based on developmental stage (Figure 3).

#### 3.1.2. Database Validation

When all spectra from adult *S. mansoni* worms and eggs were compared to the commercially available Bruker MALDI-TOF database (i.e., BDAL) for bacteria, fungi, and mycobacteria, none of the samples could be identified, thus indicating no contamination.

When raw spectra used to create the new MSPs were compared to the expanded in-house *S. mansoni* database during internal validation, all spectra were identified with LSVs ≥ 2.0, indicating a reliable identification at the species level. The average LSV score was 2.79 for adult worms and 2.56 for eggs (Table 1 and Table 2).

Validation tests, during which spectra from a subset of new independent samples were compared to the existing in-house database, revealed an identification rate of 68.9% (95% CI: 58.3–78.2%) at the genus level (LSV ≥ 1.7) and 47.8% (95% CI: 37.1–58.6%) at the species level (LSV ≥ 2) for adult *S. mansoni*, with an average LSV of 1.76. After expansion of the database (i.e., adding 12 new MSPs from adults), we obtained 100% (95% CI: 96.0–100%) correct identification at the genus level and 98.9% (95% CI: 94.0–100%) at the species level with an average LSV of 2.47 (Table 3). The increase in correct identification after database expansion was statistically significant (Fisher’s exact test, *p* < 0.001).

As for the isolated egg samples, 98.3% (95% CI: 91.1–100%) identified with LSVs ≥ 1.7, whereas 90% (95% CI: 79.5–96.2%) were identified with LSVs ≥ 2, showing an average score of 2.2 (Table 3).

#### 3.1.3. Analysis of Stool Samples Spiked with Schistosoma Mansoni Eggs

**Figure 4:**
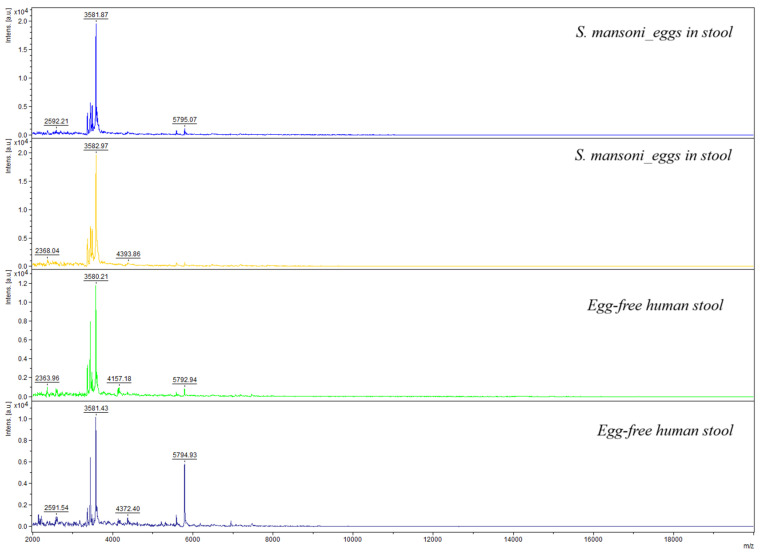
MALDI-TOF MS spectra from stool samples (egg-free stool vs. eggs spiked in stool).

When comparing raw spectra originating from the thirty-five stool samples spiked with eggs to the in-house database, none of the samples could be identified with an LSV of 1.7 or higher. Despite a 98.3% identification rate for purified eggs, analysis of 35 egg-spiked stool samples yielded no identifiable spectra. A visual representation of the spectra is displayed in Figure 4. Of note, a closer look at the most prominent peaks shows that the negative control spectra show some of the same peaks with high intensities as the spectra stemming from egg-spiked stool samples, indicating that characteristic egg-specific peaks were obscured in the spiked stool spectra, making identification impossible.

## 4. Discussion

In this study, we evaluated the capacity of MALDI-TOF MS to identify adult *S. mansoni* worms and eggs. Our results show that the expansion of the existing database (containing *S. mansoni* adult male and female spectra) by adding new MSPs led to 100% correct identification rate at the genus level (LSV ≥ 1.7) and 98.9% at the species level (LSV ≥ 2.0) for adult worms. Our database already contained adult MSPs (total < 10). The addition of 12 MSPs led to an augmentation of correct identification rates by over 30% (68.9% to 100% at the genus level). Furthermore, the decrease in the standard deviation after expansion of the database indicates an improvement in the measurement precision and consistency. MALDI-TOF MS database expansion is crucial for improving identification results. Expanding this database serves several purposes: it (i) improves spectral diversity and robustness, (ii) allows assessment of biological variables such as sex-specific protein profiles, and (iii) provides a necessary reference framework for future studies investigating whether circulating parasite-derived proteins might be detectable in alternative matrices such as serum or urine.

As stated in our previous work [28], adult *Schistosoma* worms are not accessible in infected definitive hosts and therefore do not represent a direct diagnostic target. However, they are used as reference material to evaluate the technical feasibility of MALDI-TOF MS as an identification tool and to generate a foundational spectral database. Further studies should validate the database using clinically relevant samples and optimize the protocol for direct application with biological samples (e.g., stool).

For isolated eggs, it has been shown that after specific MSPs were created and added to the database, validation tests achieved a correct identification rate of 98.3% at the genus level and 90% at the species level. However, MALDI identification of eggs in stool samples did not yield any positive results. The preparation of the spiked stool samples is an experimental approach under controlled conditions to assess the feasibility of MALDI-TOF MS for the identification of *S. mansoni* eggs. The samples do not reflect the complexity of stool samples. Despite these simplified conditions, the experiment did not reveal highly reproducible and specific spectra for a reliable identification of *S. mansoni* using stool. The failure to identify eggs in stool underscores technical limitations, where protein signals from stool debris dominate MALDI-spectra and obscure egg-derived signals, even when purified eggs are present. This highlights the current incompatibility of MALDI-TOF MS with complex biological matrices such as feces. Additionally, the need for stool debris removal prior to MALDI-TOF analysis represents a tremendous challenge, as any concentration or purification step required to enable MALDI detection would largely mirror existing microscopy-based techniques, thereby negating the principal advantages of MALDI-TOF in speed and simplicity. This substantially limits the techniques’ current diagnostic added value for intestinal schistosomiasis.

MALDI-TOF MS is now known to be the gold standard diagnostic tool for bacterial and fungal identification in high-income countries [16,31], whereas parasite identification mostly relies on microscopic examination. This method is cost-effective but is limited by being time- and resource-consuming and requires experts for diagnosis. Immunodiagnostics as well as molecular methods have been developed and generally show a higher sensitivity than microscopy. For example, the sensitivity of detection of *S. mansoni* in stool samples has been increased by PCR from 33.7% to 48.8%. In addition, PCR performed on serum samples confirmed a sensitivity of 72.7%. However, approaches such as PCR are more expensive and time-consuming [10,32,33].

Various studies have examined the use of MALDI-TOF MS for identification of other organisms. Some of them have examined arthropods like ticks [18] and mosquitoes [19,20]. Other studies have analyzed helminth parasites [21] such as nematodes like *Trichuris* [26] and *Anisakis* [34], as well as trematodes (e.g., *Fasciola* spp.) [23] and cestodes (e.g., *Taenia saginata*) [22]. Not only adult specimens, but developmental stages such as larvae were also examined. In 2019, Huguenin et al. showed that trematode cercariae can be reliably detected by MALDI-TOF MS [35]. Yet, to the best of our knowledge, this study is the first to prove the capability of MALDI-TOF MS to correctly identify isolated trematode eggs after creating reference spectra. However, compared to the established approaches (i.e., low-cost microscopy, serology, or DNA-based assays), current applications of MALDI-TOF MS do not show superiority in terms of feasibility and cost-effectiveness for schistosomiasis diagnosis using stool as biological material.

Our study has several limitations. All *Schistosoma* samples used for the current investigation have the same origin (single laboratory-adapted NMRI strain) and were all isolated from animal hosts (experimental mice), meaning that the database’s performance against genetically diverse field isolates from human hosts is unknown and requires further validation with field isolates from various origins and diverse geographical locations. Also, we could not identify spectra of *S. mansoni* eggs spiked in stool samples, which would be required for potential use as a diagnostic tool in clinical practice, as eggs are the diagnostic stage expected in stool samples, and a method of separating the eggs from the stool debris would be needed. Moreover, the negative stool samples originated from endemic regions only and were tested with microscopy-based methods. The addition of field isolates stemming from different geographic locations and different hosts, including non-endemic regions and testing them using molecular-based methods for species confirmation, would be desirable in future investigations. Further studies could aim (1) to develop optimized protocols that can be applied to alternative biological materials such as the blood, serum or urine (for *S. haematobium*) of infected humans for identification of *Schistosoma* species directly in biological samples. Therefore, MALDI-TOF MS analysis could be performed on *Schistosoma*-positive and -negative blood/sera, respectively, and combined with machine learning algorithms, differences between the two groups could be revealed, and support pattern recognition, particularly if low parasite-derived signals are present but masked by host-derived proteins, hence, help to distinguish infected from non-infected human blood/serum, as previously reported for *Plasmodium falciparum* infection [36]. Further studies could also aim (2) to add enzymatic digestion before analysis to reduce complex protein backgrounds and enhance detection of parasite-specific peptides, as MALDI-TOF MS can identify proteins and peptides. Hence, protein fragments stemming from extracted peptides can be detected by MALDI-TOF MS. Protocols using, e.g., trypsin for protein digestion before subjecting the sample to MALDI-TOF MS have been developed and might warrant further investigation in the context of schistosomiasis [37].

## 5. Conclusions

Our study demonstrates that MALDI-TOF MS shows the ability to identify adult *S. mansoni* worms and isolated eggs when high-quality reference spectra are available. This is, to our knowledge, the first study to confirm that *S. mansoni* isolated eggs can reliably be identified by MALDI-TOF MS under controlled laboratory conditions. However, our study also showed that MALDI-TOF MS is currently not suitable for direct detection of *S. mansoni* eggs in stool samples as none of the egg-spiked stool samples yielded identifiable spectra. Rather than being immediately applicable as a complementary or alternative tool in parasitological diagnostics, MALDI-TOF MS should be considered as a research technology with potential future applications that depend on substantial methodological innovation and improvements.

## Figures and Tables

**Table 1:** Internal validation: identification of spectra of 12 *Schistosoma mansoni* adult worms by MALDI-TOF MS using an in-house *Schistosoma mansoni* database.

Species	Sex	Number of Samples	Number of Spectra	Average Score (±SD)
*Schistosoma mansoni*	female	4	102	2.79 (±0.02)
	male	4	98	2.78 (±0.03)
	Mixed (male + female)	4	102	2.80 (±0.08)
	Total	12	302	2.79 (±0.02)

**Table 2:** Internal validation: identification of 11 *S. mansoni* egg samples by MALDI-TOF MS using an in-house *Schistosoma mansoni* eggs database.

Species	Protocol Used	Number of Samples	Number of Spectra	Average Score (±SD)
*Schistosoma mansoni* (eggs)	Protocol 1	3	83	2.41 (±0.04)
	Protocol 2	3	90	2.50 (±0.02)
	Protocol 3	5	143	2.69 (±0.05)
	Total	11	316	2.56 (±0.13)

**Table 3:** External validation: identification of 90 adults and 60 eggs of *S. mansoni* by MALDI-TOF MS using an in-house *S. mansoni* database before and after expansion.

Species	Number of Samples	Identification (LSV ≥ 1.7)	Identification (LSV ≥ 2)	Average Score (±SD)
Adult worms (initial database)				
*Schistosoma mansoni*	90	62/90 (68.9%)	43/90 (47.8%)	1.76 (±0.39)
Adult worms (expanded database)				
*Schistosoma mansoni*	90	90/90 (100%)	89/90 (98.9%)	2.47 (±0.12)
Eggs (expanded database)				
*Schistosoma mansoni*	60	59/60 (98.3%)	54/60 (90%)	2.2 (±0.17)

## Data Availability

The data presented in this study are available on reasonable request from the corresponding author.

## Abbreviations

The following abbreviations are used in this manuscript:

NTD	Neglected tropical disease
MALDI-TOF MS	Matrix-assisted laser desorption/ionization time-of-flight mass spectrometry
MS	Mass spectrometry
LSV	Log score value
CI	Confidence interval
CCA	Circulating cathodic antigens
HCCA	a-Cyano-4-hydroxycinnamic acid
MSP	Main spectra profile
